# Impact of the Diet on the Mortality and on Gene Expression of the Antimicrobial Peptide Tenecin 3 in *Tenebrio molitor* Larvae Infected by *Beauveria bassiana*

**DOI:** 10.3390/insects14040359

**Published:** 2023-04-04

**Authors:** Valentina Candian, Rosemarie Tedeschi

**Affiliations:** Dipartimento di Scienze Agrarie, Forestali e Alimentari (DISAFA), University of Torino, Largo P. Braccini 2, 10095 Grugliasco, Italy

**Keywords:** brewers’ spent grain, wheat bran, immune responses, entomopathogenic fungus

## Abstract

**Simple Summary:**

With the growing interest in insect rearing for feed and food use, there has been an increased focus on the health of the insects for both farm business aspects and the safety of end consumers. Recent research has highlighted how the insect immune system can be modulated by the diet. We studied the survival of yellow mealworm beetle larvae infected with an entomopathogenic fungus and the gene expression of a specific antimicrobial peptide with antifungal action in insects fed two different diets. In our trials, the partial substitution of a wheat bran-based diet by brewers’ spent grain, characterized by a high protein value, did not increase the survival rate of infected larvae but increased the expression of the antifungal peptide, depending on the timing of administration. The possibility of modulating farmed insect health through the diet, using agri-food byproducts, opens interesting new perspectives within a circular economy scenario.

**Abstract:**

Large-scale insect rearing can be subjected to microbial infections, leading to serious economic losses. When possible, the use of antibiotics is to be avoided for insects intended as feed or food and new strategies to preserve the health of the farmed insects are required. The effectiveness of insect immune system depends on several factors, including the nutritional composition of the diet. The possibility to modulate immune responses through the diet is currently a topic of great interest from an application point of view. We evaluated the effect of two different diets on the survival rate and gene expression of the antimicrobial peptide Tenecin 3 in uninfected and *Beauveria bassiana*-infected *Tenebrio molitor* L. larvae. A wheat bran diet, mixed 50% with brewers’ spent grains, could positively influence the expression of Tenecin 3 gene when uninfected *T. molitor* is allowed to develop on such a substrate from early larval stages. Even if, in our trial, the diet with added brewers’ spent grains could not reduce the mortality of the larvae infected with *B. bassiana*, higher transcriptional levels of the antifungal peptide were observed in insects fed this diet, depending on the timing of diet administration.

## 1. Introduction

In the last years, the interest in using insects as an alternative protein source for both animal and human nutrition has increased exponentially [1]. In 2022, the number of start-ups focused on edible insect farming has been estimated to be more than 297 worldwide [2]. The global edible insect market is expected to reach a global value of nearly 8 billion USD and a volume exceeding 730,000 tons in 2030 [3]. Despite the increased interest in the developed world for insects either as food or feed and the technological advances in the insect farming sector, there are still some challenges that need to be addressed. Indeed, there are critical issues with insect health that require attention, especially in large-scale insect production. In this context, insects could have reduced fitness, reproductive capacity and survival due to high population density, sub-optimal abiotic factors, poor hygiene, limited space and excessive inbreeding. Moreover, insects are more prone to viral, bacterial and fungal infections [4,5,6,7,8], which can result in increased insect mortality and disorders, as well as the death of the entire colony [9]. Even if, in case of diseases caused by different entomopathogenic microorganisms, curative treatments with antibiotics could be effective, any reduction or avoidance of antibiotic use is beneficial in light of rising global resistance. Therefore, it is necessary to define alternative strategies in order to preserve insect health in mass rearing. 

The immune system of insects is characterized by a set of mechanisms that allow protection against foreign substances and pathogen invasion. The protection against pathogens begins primarily with different barriers such as the cuticle, the intima of trachea and gut lining, tissues that are difficult to be penetrated [10,11]. Pathogens that overcome these first lines of defense subsequently face the cellular and humoral responses of the insect immune system. These elements can act cooperatively during immune responses, revealing a complex level of interaction [12]. Cellular responses are mediated by hemocytes involved in phagocytosis, encapsulation and nodulation of foreign organisms [12,13,14,15,16,17,18,19,20,21]. The majority of hemocytes circulate freely within the hemolymph (circulating hemocytes), but a significant number can be found associated with internal organs (e.g., fat body, gut, or dorsal vessel) (sessile hemocytes) [12,22,23]. The types of hemocytes and their abundance may vary according to the insect species as well as their developmental and physiological state [12,16,22,23,24,25,26]. Humoral events, on the other hand, include the production and release into the hemolymph of antimicrobial peptides (AMPs), activation of prophenoloxidase (proPO) and production of reactive oxygen and nitrogen compounds (ROS and RNS, respectively) [10,15,16]. A variety of AMPs have been isolated and characterized in insects to date and have gained a particular interest for their useful potential applications in pharmaceutical and agricultural fields [27].

The effectiveness of the insect immune system can vary depending on biotic and abiotic factors including temperature [28,29,30,31], population density, age of the individual and the interaction with pathogens. The type of feeding substrate and its availability also profoundly affect the insect immune responses as they require metabolic resources. Under conditions of nutritional deficiencies, individuals experience difficulties in implementing immune responses [32]. It has been shown that, to compensate for the energy expenditure following an immune challenge, insects may ingest up to five times more food per day [33]. The nutritional composition of the diet, in particular the protein/carbohydrate ratio, is also an important factor. The effect of the diet on insect immune responses has recently been highlighted [33,34,35,36,37,38,39]. Nutrient-poor diets and non-optimal protein–carbohydrate ratios result in a lower production of AMPs and hemocytes and a decrease in the insect endogenous bacteria [36,40,41]. The possibility to modulate both cellular and humoral immune responses of insects through the diet is a very topical subject [38,41]. However, the diet-mediated effect on insect immunity is still poorly investigated. Moreover, research is mainly focused on the evaluation of the insect immune challenge with Gram-positive and Gram-negative bacteria. 

The possible influence of entomopathogenic fungi on the insect humoral responses has hardly been investigated. Specifically, the temporal expression of genes’ encoding for AMPs in the hours following pathogen attack has only been marginally evaluated and no study has ever simultaneously examined the possible influence of the rearing diet. Our study aims to evaluate the effect of two different diets on the survival rate and expression level of the gene encoding for the Tenecin 3, an AMP with antifungal activity [42], in uninfected and *Beauveria bassiana*-infected larvae of *Tenebrio molitor* L. (Coleoptera: Tenebrionidae). 

## 2. Materials and Methods

*Tenebrio molitor* larvae used in the present study originated from mass rearings maintained on a wheat bran diet at the Unit of Entomology of the Department of Agricultural, Forest and Food Sciences (DISAFA; University of Torino, Grugliasco, Italy). In uninfected and *B. bassiana*-infected larvae, we looked at the effect of the diet on *T. molitor* survival and Tenecin 3 encoding gene expression at two timings of diet administration. All rearings were maintained in a climate-controlled chamber (T: 25 °C; 16:8 h L:D photoperiod). 

### 2.1. Diets and Feeding Times

Experimental insects were reared on two different diets: (i) 100% wheat bran diet (BRAN) (raw fibre 11.50%, moisture 15.50%) [Perazzone S.r.l., Cerrione, Italy], used as a control, and (ii) a diet composed of 50% wheat bran and 50% dried brewers’ spent grain (+GRAIN) (barley malt) [Microbirrificio Birra Elvo, Graglia, Italy]. For each diet, 2 different timings of diet administration were tested: (i) larvae maintained for 14 days on the substrate (14-day feeding) and (ii) larvae maintained directly on the substrate from their eclosion (full feeding).

In the case of the 14-day feeding, 350 14–17 mm larvae (X-XII instar) were collected from the insect mass rearing fed a wheat bran diet. Larvae were weighed, transferred to a plastic tray (24 × 17 × 12 cm) containing the BRAN or +GRAIN diet (one for each diet) and maintained for 14 days (until XIII-XIV instar was reached, 21–24 mm length), then weighed again and used in the trials. In the second case (full feeding), 100 adults collected from the mass rearings fed a wheat bran diet were placed into new plastic trays with the BRAN or +GRAIN diet (one for each diet) for one week, sufficient time to ensure mating and oviposition. Later, adults were removed and the new generation was used in the trials. Again, individuals were weighed when they reached X-XII and XIII-XIV larval instar. The average daily gain (ADG) of XIII-XIV instar larvae was evaluated according to the formula below:

ADG = (final weight − initial weight)/number of days on diet

For each diet and timing of diet administration, substrates were provided *ad libitum* and portions of carrots were added twice per week as a source of hydration. 

**Figure 1:**
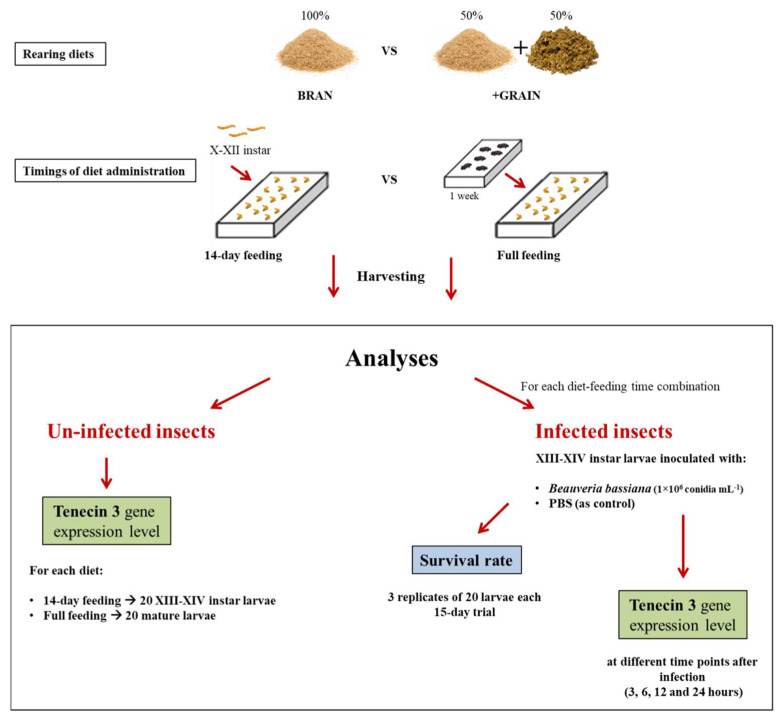
Experimental design and description of the analyses preformed on uninfected and infected insects. *Tenebrio molitor* larvae or adults were reared on BRAN or +GRAIN diet for different timings of diet administration. Groups of X-XII instar larvae were collected and reared on the tested diets for 14 days prior to being used in the trials (14-day feeding) or adults were allowed to oviposit directly on the tested substrates and the new generation was used in the trials (full feeding). Depending on the trial, different instars of *T. molitor* larvae were analyzed. In the first trial, Tenecin 3 expression level was evaluated in XIII-XIV and mature uninfected larvae were fed the different diets for 14-day feeding or a full feeding period. Subsequently, for each diet and feeding time combination, groups of XIII-XIV larvae were inoculated by injection with *B. bassiana* or PBS, as control. Two trials were performed. In the first one, the survival rate of infected insects was assessed in a 15-day trial. In the second one, Tenecin 3 transcriptional level was evaluated in groups of larvae collected 3, 6, 12 and 24 h after injection.

Three different trials were performed. A first trial was set up in order to evaluate the Tenecin 3 transcriptional level in uninfected larvae fed different diets (BRAN or +GRAIN) for different timings of diet administration (14-day or full feeding). Two other trials were set up in order to assess the survival rate of *B. bassiana*-infected insects previously reared on different diets (BRAN or +GRAIN) for different timings of diet administration (14-day or full feeding) (second trial) and their Tenecin 3 encoding gene expression level (third trial) at different time points after infection (Figure 1). 

### 2.2. Tenecin 3 Gene Expression Analysis in Uninfected Tenebrio Molitor Larvae

XIII-XIV instar larvae collected at the end of the 14-day feeding period and mature larvae maintained on the diet since their eclosion (full feeding) were used in order to assess the expression of the Tenecin 3 encoding gene in uninfected *T. molitor*. For each diet (BRAN and +GRAIN) and feeding time combination (14-day and full feeding), 20 larvae were sampled and analyzed. Mature larvae were collected when the first two pupae were observed in the rearing.

#### 2.2.1. RNA Extraction

Larvae were washed in diethylpyrocarbonate (DEPC) water [Merck KGaA, Darmstadt, Germany], 75% ethanol in DEPC water and DEPC water for 30 s, with the aim of removing any diet residues and any other possible contaminants present (e.g., insect frass, bacteria). Collected individuals were stored in NucleoProtect^®^ RNA [Macherey-Nagel^™^, Düren, Germany] at −20 °C until further analysis. Insects were grounded individually with liquid nitrogen and total RNA extraction was performed with the “SV Total RNA Isolation System” [Promega, Madison, WI, USA] according to the manufacturer instructions. RNA quality and concentration were assessed with an ND-1000 spectrophotometer [NanoDrop Technologies, Wilmington, DE, USA]. Subsequently, 1 μg of RNA was used for cDNA synthesis by iScriptTM cDNA Synthesis Kit (Bio-Rad, Hercules, CA, USA). Then, the cDNA was diluted (1:10) and used in quantitative real-time PCR (qPCR) in order to assess the AMP encoding gene expression level.

#### 2.2.2. Quantitative Real-Time PCR

Reactions were performed with SensiMixTM SYBR^®^ No-Rox kit [Bioline Meridian Bioscience, London, UK] using primers targeting the Tenecin 3 (Forward: 5′-GATTTGCTTGATTCTGGTGGTC-3′ and Reverse 5′-CTGATGGCCTCCTAAATGTCC-3′) [43]. The reference gene was 18S RNA coding gene (Forward: 5′-TTCGAGCAGGAAATGGCCAC-3′ and Reverse 5′-TTGGAAGAGAGCCTCTGGAC-3′) [44]. Analyses were conducted in clear HardShell^®^ Low-Profile 96-Well PCR Plates (Bio-Rad, Hercules, CA, USA) with a 50 μL mixture containing 25 μL of SYBER^®^ Green, 0.5 μL of each primer (25 µM), 5 µL of cDNA sample and 19 μL of sterile H_2_O, sealed with adhesive Microseal^®^ PCR Plate Sealing Film (Bio-Rad, Hercules, CA, USA); samples were analyzed in triplicate. The analysis was performed on a CFX ConnectTM Real-Time PCR Detection System (Bio-Rad, Hercules, CA, USA), applying the following thermic protocol: 95 °C for 10 min, followed by 40 cycles of 95 °C for 15 s, 58.5 °C for 15 s and 72 °C for 15 s. A final step for melting curve analysis from 58.5 to 95 °C, measuring fluorescence every 0.5 °C, was added. Results were analyzed using the CFX ManagerTM Software (Bio-Rad, Hercules, CA, USA) for Ct determination. Relative quantification of target genes was calculated using the 2^−ΔΔCt^ method [45] and expressed as a fold change. 

### 2.3. Tenebrio molitor Infection Assays with Beauveria bassiana

For each diet (BRAN or +GRAIN) and feeding time combination (14-day or full feeding), XIII-XIV instar larvae were collected and used in order to assess the survival rate and Tenecin 3 transcriptional level in *B. bassiana*-infected *T. molitor*.

#### 2.3.1. Inoculum Preparation

*Beauveria bassiana* ATCC 74040 was cultured from the commercial product *Naturalis*^®^ [Biogard, CBC (Europe) S.r.l., Grassobbio, Italy] by distributing 50 µL of Naturalis^®^ on a Petri dish surface (9 cm diameter) containing 20 mL of SDYA medium (40 g dextrose, 10 g peptone, 5 g yeast extract and 15 g agar for 1L of distilled water, final pH 5.6) and periodically re-plated. *Beauveria bassiana* plates were incubated at 25 °C for 20 days until conidia appeared, which were collected using a sterile loop and transferred into 600 µL of 1× phosphate-buffered saline (PBS) [Merck KGaA, Darmstadt, Germany]. The inoculum was centrifuged at 2500 rpm for 5 min at room temperature in order to separate the mycelium from the conidia and, using a hemocytometer, the concentration of the inoculum was adjusted to 1 × 10^6^ conidia mL^−1^. The conidia germination capacity was verified by inoculating 3 plates containing 13 mL of SDYA medium with 50 µL of inoculum solution and keeping them in an incubator at 25 °C for the following days until the mycelium appearance.

#### 2.3.2. Survival Rate

For each diet (BRAN, +GRAIN), the survival rate of XIII-XIV larvae was assessed in individuals fed the diet for 14 days or in larvae eclosed directly on the tested diet (full feeding). Larvae were inoculated with 1 µL of 1× PBS, used as control or 1 µL of *B. bassiana* solution by injection in the thorax by means of a Hamilton Microliter 700 series syringe (Hamilton Company, Reno, NV, USA). Groups of 20 inoculated larvae were transferred in plastic jars (Ø 8.5 cm, height 8.5 cm) closed with a micro-perforated cloth (mesh size 0.3 × 0.4 mm) containing 60 mL of diet (BRAN, +GRAIN) and a portion of carrot. Three replicates of 20 larvae each were set up for each diet–feeding time combination, for a total of 60 individuals per each combination and inoculum (PBS, *B*. *bassiana*). Jars with inoculated larvae were kept in a climate chamber under controlled conditions (26 ± 3 °C, 50 ± 10% RH, 16:8 L:D photoperiod). The survival rate was assessed every 24 h for 15 days. Any dead individual was collected and placed in Petri dishes in the presence of moistened cotton and sealed with Parafilm^®^ M [Bemis^®^ Company, Inc., Neenah, WI, USA] in order to maintain a high humidity level and thus allow the development of the entomopathogenic fungus.

#### 2.3.3. Tenecin 3 Gene Expression Analysis in *B. bassiana*-Infected *T. molitor* Larvae

For each diet-feeding time combination, 60 XIII-XIV instar larvae were inoculated with 1 µL of PBS 1× or 1 µL of the *B. bassiana* solution following the protocol already described for the survival assays. Larvae were maintained in plastic jars (Ø 8.5 cm, height 8.5 cm) (one for each diet-feeding time combination) closed with a micro-perforated cloth (mesh size 0.3 × 0.4 mm) and containing 60 mL of the tested diet (BRAN, +GRAIN) and a portion of carrot. When possible (due to larval mortality), 10 larvae were collected for each diet–feeding time combination on the tested diet 3, 6, 12 and 24 h after injection. Then, insects were immediately washed in sterile water treated with DEPC (30 s), 75% EtOH (30 s) and then water with DEPC (30 s) in order to remove any diet residues and any other possible contaminant present (e.g., insect frass, bacteria) before being stored at −20 °C in NucleoProtect^®^ RNA. The expression of the gene encoding for Tenecin 3 was assessed following the protocol previously described (Section 2.2.1 and Section 2.2.2).

### 2.4. Statistical Analysis

Statistical analyses were performed using SPSS Statistics 29 (IBM Corp., released 2017, Armonk, NY, USA) and outcomes were considered significant at *p* < 0.05. Gene expression data were subjected to logarithmic (log10) transformation for normality before statistical analysis. Gene expression data of uninfected *T. molitor* larvae were subject to pair-wise comparison of the mean with Student’s *t*-test. In the case of infected larvae, two statistical analyses were performed. First of all, AMP expression data were analyzed for each time of diet administration in order to compare the expression level of the Tenecin 3 in PBS- and *B. bassiana*-infected larvae at different time points after the injection. In this case, AMP expression level data were analyzed using a generalized linear model (GLM) applied with a normal distribution. Averages were separated by Bonferroni correction. Subsequently, in order to compare the expression level of the Tenecin 3 at different time points in *B. bassiana*-infected larvae fed the +GRAIN diet for 14 days or since their eclosion (14-day feeding, full feeding), data were subject to pair-wise comparison of the mean using Student’s *t*-test. A 15-day survival analysis was performed with PBS- and *B. bassiana*-injected larvae with censored cases using the Kaplan–Meier method. Significant differences among each inoculum–diet–feeding time combination were determined with the log-rank test (Mantel–Cox). The effect on larval survival under different diet (BRAN, +GRAIN), time of diet administration (14-day feeding, full feeding) and inoculum (PBS, *B. bassiana*) conditions was determined by Cox regression analysis.

## 3. Results

### 3.1. Tenebrio molitor Growing Performances

The average weight of X-XII instar larvae reared on wheat bran and then used for the 14-day feeding period trial was 0.0422 g. After the 14-day feeding period, an ADG of 0.0033 g (larval mean weight of 0.0884 g) was recorded with the BRAN diet and of 0.0036 g (larval mean weight of 0.0926 g) with the +GRAIN diet. In the case of larvae eclosed directly on the tested diet, the average weight of X-XII instar larvae was 0.0424 and 0.0464 g with the BRAN and the +GRAIN diet, respectively. An ADG of 0.0028 g (larval mean weight of 0.0811 g) was recorded with the BRAN diet and of 0.0035 g (larval mean weight of 0.0913 g) for the +GRAIN diet.

### 3.2. Tenecin 3 Gene Expression Analysis in Uninfected Tenebrio molitor Larvae

**Figure 2:**
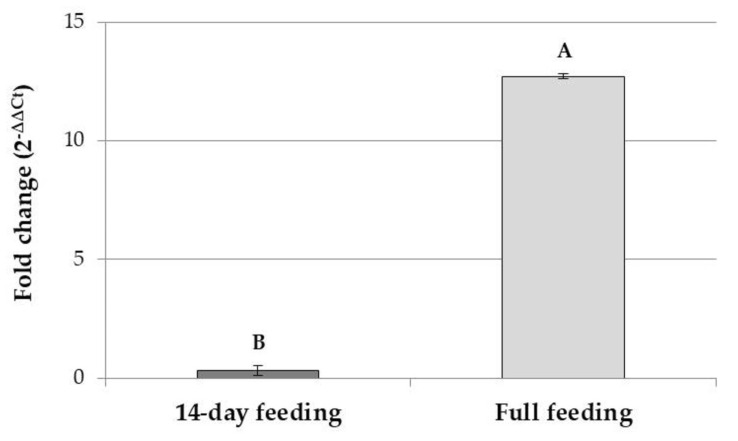
Gene expression (2^−∆∆Ct^) of Tenecin 3 in *T. molitor* larvae reared on +GRAIN after a 14-day feeding period or in mature larvae eclosed directly on +GRAIN and collected when the first two pupae were observed in the rearing. Values are reported as average fold change variation (mean ± SE). Samples were normalized against larvae reared on BRAN for their respective timescales. Different letters indicate significantly different values (Student’s *t*-test, *p* < 0.05).

Significant differences depending on the timing of diet administration were observed for Tenecin 3 encoding gene expression level (Student’s *t*-test: *t* = −3.179; *df* = 16.130; *p* = 0.006). The higher values were recorded with larvae fed the +GRAIN diet from their eclosion. A down-regulation of the AMP encoding gene (fold change 0.32) was observed in larvae fed the +GRAIN diet for a 14-day feeding period compared to larvae fed the BRAN diet for the same period (Figure 2). In individuals eclosed directly on the +GRAIN diet, an up-regulation of the Tenecin 3 encoding gene was observed (fold change 12.37) compared to larvae fed a BRAN diet since their eclosion (Figure 2).

### 3.3. Tenebrio molitor Infection Assays with Beauveria bassiana

#### 3.3.1. Survival Rate

**Figure 3:**
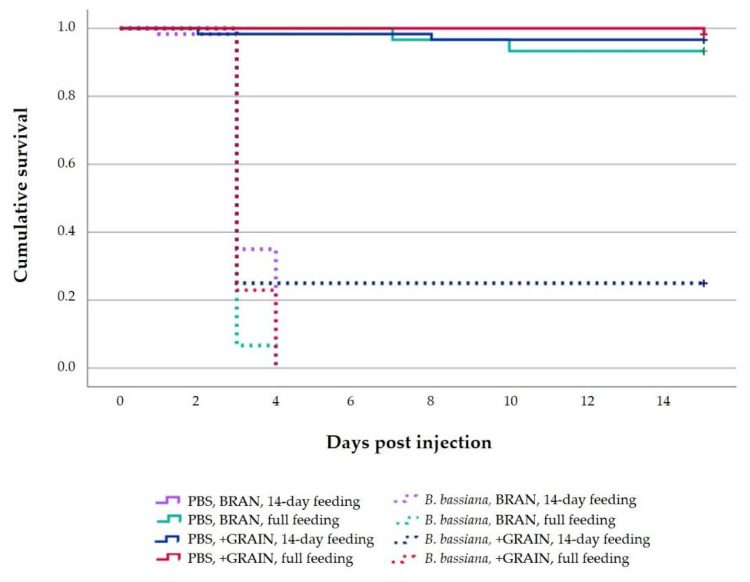
Survival curve according to Kaplan–Meier analysis of larvae fed different diets (BRAN, +GRAIN) for different timings of diet administration (14-day feeding, full feeding) after injection with PBS or *B. bassiana*. The survival lines of “PBS, BRAN, 14-day feeding” and of “PBS, +GRAIN, full feeding” coincide perfectly. Therefore, only the “PBS, +GRAIN, full feeding” line is evident. Censored cases are indicated by a cross.

The survival curves of PBS- (control groups) and *B. bassiana*-injected larvae are reported in Figure 3. According to the log-rank test analysis, significant differences were found among each inoculum–diet–feeding time combination (log-rank test: df = 7; *χ*^2^ = 464.326; *p* < 0.001). The highest survival rate was recorded in larvae treated with PBS independently of the diet and the timing of diet administration. All *B. bassiana*-infected individuals died within 4 days after injection, with the exception of larvae fed a +GRAIN for 14 days. In this case, 75% of the larvae died 3 days after the injection. The Cox regression analysis revealed statistically significant differences (df = 1; *χ*^2^ = 22.044; *p* < 0.01) between PBS- (control groups) and *B. bassiana*-infected larvae due to the inoculum used in the trials. Statistical differences were not found for the diet and timing of diet administration nor factors’ interactions. 

#### 3.3.2. Tenecin 3 Gene Expression Analysis in *B. bassiana*-Infected *T. molitor* Larvae

**Figure 4:**
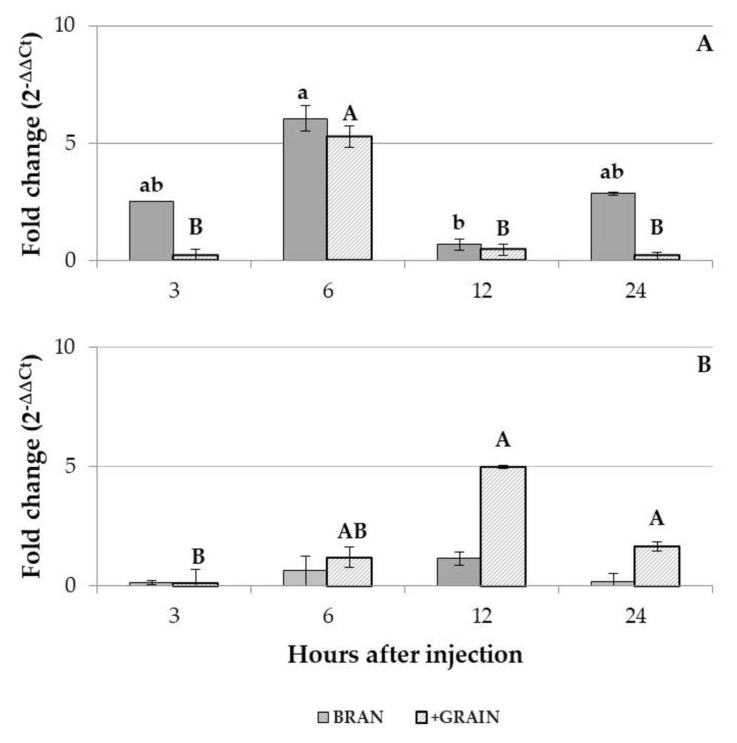
Gene expression (2^−∆∆Ct^) of Tenecin 3 after 3, 6, 12 and 24 h from *B. bassiana* injection in *T. molitor* larvae reared on BRAN and +GRAIN for a 14-day feeding period (**A**) and diet since their eclosion (full feeding) (**B**). Values are reported as average fold change variation (mean ± SE). For each time point, samples were normalized against larvae injected with PBS and reared on BRAN or +GRAIN for a 14-day or diet since their eclosion, respectively. Different letters indicate significantly different values (GLM, *p* < 0.05).

After the 14-day feeding period, significant transcriptional differences were recorded in *B. bassiana*-infected insects fed the BRAN (GLM: gl = 3; *χ*^2^ = 7,846; *p* = 0.049) and the +GRAIN diet (GLM: df = 3; *χ*^2^ = 22.887; *p* < 0.001) at different time points after injection compared to larvae injected with PBS. In larvae fed the BRAN diet, the up-regulation of the Tenecin 3 gene expression was observed 3, 6 and 24 h after *B. bassiana* injection (fold change: 2.53; 6.06 and 2.86, respectively) while a down-regulation of the AMP coding gene expression level was recorded 12 h after *B. bassiana* injection (fold change: 0.68) compared to larvae injected with PBS (Figure 4). In larvae fed the +GRAIN diet, the up-regulation of the Tenecin 3 was observed 6 h after *B. bassiana* injection (fold change: 5.28) while the down-regulation of AMP gene was observed at 3, 12 and 24 h after treatment (fold change: 0.16; 0.45 and 0.19, respectively) compared to larvae injected with PBS (Figure 4A). 

In the case of larvae eclosed directly on the tested diet, significant AMP transcriptional differences at different time points after injection were observed only in insects fed the +GRAIN diet (GLM: df = 3; *χ*^2^ = 14.240; *p* = 0.003) (Figure 4B). In insects fed the BRAN diet, a down-regulation of Tenecin 3 encoding gene was observed 3, 6 and 24 h after *B. bassiana* injection (fold change: 0.13, 0.61 and 0.18, respectively) while a slight up-regulation was recorded 12 h after injection (fold change: 1.13). In the case of larvae eclosed directly on the +GRAIN diet, an up-regulation of Tenecin 3 was observed 6, 12 and 24 h (fold change: 1.20, 4.98 and 1.64, respectively) after *B. bassiana* infection, while the AMP down-regulation was recorded 3 h after injection (fold change: 0.10) (Figure 4B).

**Figure 5:**
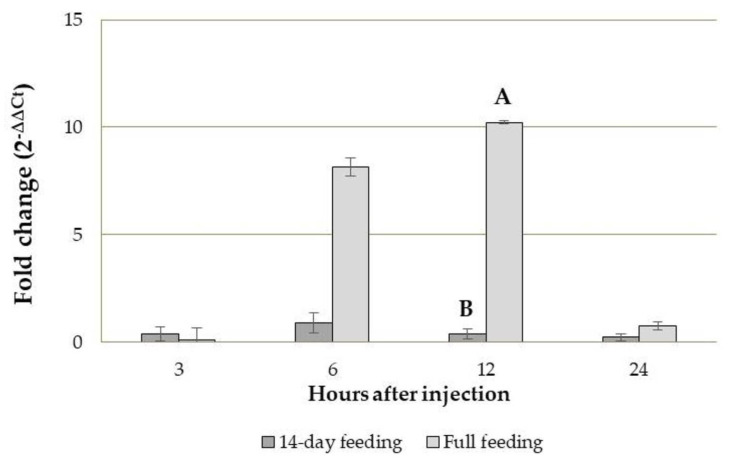
Gene expression (2^−∆∆Ct^) of Tenecin 3 after 3, 6, 12 and 24 h from *B. bassiana* injection in *T. molitor* larvae reared on +GRAIN for a 14-day feeding period or diet since their eclosion (full feeding). Values are reported as average fold change variation (mean ± SE). For each time point, samples were normalized against larvae injected with *B. bassiana* and reared on BRAN for a 14-day feeding period or since their eclosion (full feeding), respectively. Different letters indicate significantly different values (Student’s *t*-test, *p* < 0.05).

Moreover, the expression level of the Tenecin 3 in *B. bassiana*-infected larvae fed the +GRAIN diet for different feeding periods (14-day feeding, full feeding) was compared with the one of *B. bassiana*-infected larvae fed the BRAN diet for the same time. Significant differences, depending on the feeding period, were observed 12 h after injection for Tenecin 3 encoding gene expression level (Student’s *t*-test: *t* = 5.000; *df* = 4; *p* = 0.002). No differences were found 3, 6 and 24 h after the injection. In general, in insects fed the +GRAIN diet and infected with *B. bassiana*, a down-regulation of Tenecin 3 encoding was recorded 3, 6, 12 and 24 h after infection (fold change: 0.39, 0.89, 0.39 and 0.22, respectively) compared with larvae fed the BRAN diet for a 14-day feeding period and inoculated with *B. bassiana* (Figure 5). In the case of larvae eclosed directly on the +GRAIN diet and infected with *B. bassiana*, a down-regulation of Tenecin 3 encoding was recorded 3 and 24 h after infection (fold change: 0.09 and 0.75, respectively) while an up-regulation of the AMP encoding gene was observed 6 and 12 h after infection (fold change: 8.13 and 10.22, respectively) compared with larvae eclosed directly on the BRAN diet and infected with *B. bassiana* (Figure 5). 

## 4. Discussion

In the last decade, many studies aimed to design the best diet formulation for *T. molitor* mass rearing also considering its ability to grow on former foodstuffs, by-products and side-stream products [46,47,48,49,50,51,52]. These studies have mainly focused on the effect of by-products-based diets on the lifespan, growth, productivity and larval nutritional composition. One of the most investigated by-products for *T. molitor* mass rearing is the brewers’ spent grain, as it represents around 85% of the total by-products generated by the brewing industry [53]. 

Although evaluating changes in weight of larvae reared on different substrates was not the purpose of this research, a higher final weight was observed in XIII-XIV instar larvae reared on the +GRAIN diet both after the 14-day feeding and the full feeding period. Indeed, as previously reported, adding 50% brewers’ spent grain to a wheat bran diet helps to increase *T. molitor* larval weight and to reduce the rearing time [54]. Dried brewers’ spent grains are characterized by a high protein content, approximately twice that of a wheat bran diet [55], which positively influences insect growth rate. Moreover, protein-rich diets also lead to higher immune responses compared to diets with a higher carbohydrate content [56]. 

Recent studies have shown how the immune response mediated by AMPs could be also modulated by the rearing diet [38,39,42]. Indeed, the diet protein content directly influences the production of amino acids that can be used for the synthesis of substrates and enzymes involved in defense reactions [57]. Moreover, the diet may affect the gut microbiota which plays a substantial role also in the regulation of the immune system and in the protection against pathogens [58,59]. To date, in the case of *T. molitor*, only Tenecin 3 showed an exclusive antifungal activity and its clear antifungal effect was observed against *B. bassiana* blastospores in vitro [60]. However, the transcriptional level of this peptide is still poorly investigated and no study has ever simultaneously examined the possible influence of the rearing diet on its encoding gene expression level. 

Therefore, we assessed the transcriptional differences of this antifungal peptide in mature and XIII-XIV larvae fed a +GRAIN diet. Compared with a wheat bran diet, the +GRAIN diet positively increases the transcription of the Tenecin 3 in mature larvae when *T. molitor* develop on this substrate from early larval stages. In contrast, a down-regulation of the gene was observed in XIII-XIV instar larvae that fed on the diet for only 14 days. However, it is essential to remember that insect immune responses can be plastic with the development [61,62]. The overall insect immune responses could vary with the insect growth and within the same instar in a pattern, which seems mostly related to the species [63]. For example, stronger cell-mediated and humoral immune responses were observed at the beginning of the last larval instar of *Manduca sexta* L. (Lepidoptera: Sphingidae) [61] and *Bombyx mori* L. (Lepidoptera: Bombycidae) [62]. In contrast, higher humoral immune responses were recorded at the end of the last larval instar of *Galleria mellonella* L. (Lepidoptera Pyralidae) [64] and *Ostrinia furnacalis* (Guenée) (Lepidoptera: Crambidae) [65]. To the best of our knowledge, similar studies were never performed on *T. molitor*. Overall, no studies identified precisely when immune responses start to change during insect development and the possible influence of the rearing diet on immune responses has never been taken into account. Because of the importance of insects as food and feed, understanding how immunity varies throughout development is critical and this should not be overlooked in large-scale insect production.

The quality of the diet provided to insects’ early developmental stages also seems to largely affect the growth rate and immune responses of the later developmental stages [66,67]. For these reasons, it should be taken into account that, prior feeding +GRAIN diet for 14-day, insects were reared on a wheat bran diet. We are aware that this may have affected the immune system. However, our intention was to assess the feasibility of subjecting the larvae to a sudden change of diet to strengthen the immune system against *B. bassiana* infection. In our study, only the type of the inoculum (PBS, *B. bassiana*) influenced the survival rate, while the feeding period and the diet did not affect it. All dead larvae previously injected with *B. bassiana* were totally covered by the mycelium 7 days after the injection. All *B. bassiana*-infected individuals eclosed directly on the tested substrate died 3–4 days after the fungus injection. Even if no significant differences have been recorded for timing of diet administration, a higher survival rate was observed when *B. bassiana*-infected larvae were previously maintained on +GRAIN diet for the 14-day feeding period. In this case, 25% of the infected larvae survived, even if seriously debilitated.

Different factors may have led to this result. The insect survival could be related to the sudden stimulation of the immune system due to the inclusion of a higher protein substrate into the diet for a 14-day period. In addition, a sudden change of diet could affect the gut microbiota. The impact of the diet on the gut microbiota profile and composition is an accepted and well-studied phenomenon in different insect species [37,52,68]. It may lead to alterations in the susceptibility to *B. bassiana* or to substantial modifications in the development of diseases. Indeed, many insects utilize symbiotic bacteria in conjunction with the immune systems to cope with antagonists [69]. Otherwise, feeding a +GRAIN diet for a 14-day period could have influenced the transcription of other AMPs or other insect immune responses effective against *B. bassiana* infection. Indeed, during an immunity challenge, insect hosts conjunctively employ innate immunity responses [70] and their synergistic interactions can lead to a broad-spectrum host response. It has been already recorded that, following the infection with *B. bassiana*, Tenecin 1, 2 and 3 showed to be up-regulated in *T. molitor* larvae [71]. Moreover, in our study, insects were infected by injection in order to highly stimulate the insect immune system. Probably, the higher concentration of the conidia solution and its injection directly in the hemocoel led to a different mortality level compared to the one obtained by Maistrou et al. [60] where larvae were infected applying a droplet of conidia solution on the intersegmental membrane. The stimulation of immune responses by diet in a normal situation of active entry by the fungus still needs to be investigated. Perhaps, in this situation, the immune responses have more time to act and be effective. We are aware that the analysis of gene expression of a single peptide following changes in diet is insufficient to obtain a complete and detailed picture of the immune response following fungal infection. The purpose of this research is simply to highlight the possibility of modulating the immune system through variations in diet (composition and timing of administration) in order to open new perspectives on immune response modulation.

Although several studies on entomopathogenic fungus–host interactions have been conducted, the possible influence of the entomopathogenic fungus on insect humoral responses has still been hardly investigated. Specifically, the temporal expression of gene encoding for AMPs in the first few hours after pathogen infections has only been marginally considered [72] and mostly after bacterial infections. In order to understand which factors could have important roles in the AMPs’ modulation, it is important to investigate how AMP transcriptional levels vary temporally and according to the type of pathogen challenge received. For example, the expression of AMPs genes in *Drosophila* begins 1–3 h after an immune challenge and reaches peaks at 3–12 h after a challenge [73]. In the hemolymph of *Pseudoplusia includens* (Walker) (Lepidoptera: Noctuidae) and *Bombus terrestris* L. (Hymenoptera: Apidae), detectable AMPs’ transcriptional levels have been recorded 2 h after an immune challenge [74,75]. The duration of antimicrobial activity in the hemolymph can be long. In *T. molitor* exposed to spores of *Metarhizium anisopliae*, the antimicrobial response is maintained for at least 7 days [76].

In our trials, *B. bassiana* infection determined an up-regulation of Tenecin 3 in individuals reared on both diets (BRAN, +GRAIN) compared to PBS injection, confirming that this AMP is involved in antifungal responses [41,42]. A greater up-regulation was recorded 6 h after the infection in insects fed the BRAN or the +GRAIN diet for the 14-day feeding period. In insects eclosed directly on the tested substrate, higher transcriptional level of the antifungal peptide was recorded only in larvae fed a +GRAIN diet starting from 6 h after the fungal injection. The maximum peak was observed 12 h after injection. 

When we compared the Tenecin 3 transcriptional level of infected larvae fed the +GRAIN diet with infected insects fed the BRAN diet, interesting differences emerged concerning the time of diet administration. Significant differences between the feeding period were observed only 12 h after the immune challenge. Although at 6 h, no significant differences were observed between the feeding period, an up-regulation was observed in individuals eclosed directly on +GRAIN diet. In this case, in a very small number of samples (2) analyzed for the +GRAIN diet, 21- to 65-fold upper transcript levels were observed compared to the control. Maintaining larvae on the diet since their eclosion increased the transcription of the antifungal peptide, while shorter timing negatively affected it. These results showed how the timing of diet administration influences the insects’ immune responses as already highlighted for *Hermetia illucens* (L.) (Diptera: Stratiomyidae) [38]. 

## 5. Conclusions

In large-scale insect production, greater attention is dedicated to the diet optimization in order to guarantee high insect performance and productivity and higher quality of the specific products of interest (e.g., proteins for feed, biodiesel, nutraceuticals). However, the effect of the diet on insect immune responses is an aspect still largely overlooked. In particular, modulating the transcription of AMPs through the diet is a valuable opportunity in order to optimize health status in insect mass rearing. Further investigations should not only research the best diets in order to support insects’ quality and quantity of production (e.g., protein, lipids) but should also investigate the possible impact of the diet (e.g., type of substrate, time of diet/inclusions’ administration) on the insect immune responses. Indeed, particular dietary supplements provided at critical times (e.g., particular stage transitions, reproduction) could help to optimize health status in large-scale insect production. 

## Data Availability

The data presented in this study are available from the corresponding author.
